# Effects of inclusion level of black soldier fly larvae protein or oil on broiler growth performance during heat stress

**DOI:** 10.1016/j.psj.2025.105883

**Published:** 2025-09-23

**Authors:** T. Veldkamp, A. Rezaei Far, J.J. Mes, S. Naser El Deen, P.G. van Wikselaar, I. Fodor, H. Chen

**Affiliations:** a Wageningen Livestock Research, Wageningen University & Research, De Elst 1, Wageningen, AH 6700, the Netherlands; b Wageningen Food and Biobased Research, Wageningen University & Research, Wageningen, the Netherlands; c Cargill Animal Nutrition & Health, Global Innovation Center Velddriel, Velddriel, the Netherlands

**Keywords:** Black soldier fly larvae protein, Black soldier fly larvae oil, Broiler, Heat stress, Insect

## Abstract

Beyond their nutritional value, insects contain bioactive compounds, including chitin, lauric acid, and antimicrobial peptides, and could potentially exert positive effects on animals consuming insect-based diets in health challenging conditions. A total of 648 Ross 308 male chicks were fed four different soybean meal-maize-wheat based diets in which soybean meal or oil was partly replaced by black soldier fly larvae (**BSFL**) protein or oil, respectively, in cyclic heat stress or thermoneutral (control) conditions. BSFL protein or oil was included at 5 and 10 %, and 2 and 4 %, respectively, in a 2 × 2 × 3 factorial design. The growth performance (ADFI, ADG, body weight gain : feed intake ratio (**G:F**), and mortality) was recorded at d 9, 21, and 34. Carcass yields were determined at 35 d of age and blood and ileal tissue samples were collected to examine heat stress-related biomarkers.

The heat stress challenge was successfully implemented, demonstrated by a significant reduction in ADFI, with numerical reductions in ADG and final BW across the overall trial. The cyclic heat stress applied was mild and chronic, with mortality and removals recorded at 2 %. Broilers exposed to heat stress showed elevated levels of ileum calprotectin, while serum corticosterone levels tended to be lower compared to controls.

A significant interaction was observed between heat stress, insect product, and inclusion level. In heat stress conditions, the inclusion of BSFL oil led to a numerical increase in ADFI, ADG, and BW, whereas inclusion of BSFL protein resulted in a numerical decrease in BW. BSFL protein at 5 and 10 % and BSFL oil at 2 and 4 % inclusion level did not exhibit notable growth performance benefits in Ross 308 broilers under mild cyclic heat stress conditions. The mild nature of the heat stress challenge may have limited the detection of BSFL product effects, highlighting the need for future investigations under more intense heat stress conditions.

## Introduction

In recent years, insects have received increasing attention as a sustainable source of raw materials for animal feed, particularly for fish, poultry, and swine. Utilizing insects in feed for farmed animals offers a promising alternative due to their nutritional properties and the potential environmental benefits, given the sustainability advantages associated with insect farming (Dicke, 2018). Several insect species can be efficiently reared on organic side streams and used to feed a range of animal species (Gasco et al., 2019). Overall, most edible insect species are rich in essential nutrients, including amino acids, fatty acids, minerals, and vitamins (Finke and Oonincx, 2023). In addition to their nutritional value, insects may offer additional health benefits over conventional feed sources, as suggested by existing literature. Compounds found in insects, such as chitin, lauric acid, and antimicrobial peptides, may exert beneficial effects on animals fed insect-based diets (Gasco et al., 2021; Veldkamp et al., 2022).

High environmental temperatures negatively impact the welfare, health, and productivity (Ghezzi et al., 2024) of farmed animals outdoors (Thornton et al., 2021) as well as indoors (Schauberger et al., 2020) and are considered one of the major challenges in global agriculture. Limited information exists on the effect of insect products in mitigating the adverse effects of heat stress. Heat stress is a significant source of systemic oxidative stress, causing a redox imbalance that favors prooxidants over antioxidants (Oke et al., 2024). It has been shown to negatively affect feed intake, growth performance, immune function, and oxygen levels, and contribute to increased mortality. Additionally, heat stress deteriorates meat quality in chickens (Mishra and Jha, 2019). Several studies have highlighted the antioxidant potential of insect proteins, including those from black soldier fly larvae (**BSFL**) (Huang et al., 2024; Mouithys-Mickalad et al., 2020; Zhu et al., 2020). Medium-chain fatty acids (MCFAs, C6-C12) reduce the host's inflammatory response (Carlson et al., 2015) and are potential candidates for use as feed supplements to counteract the effects of heat stress (Chen et al., 2022; Santos et al., 2019). Insect oil derived from BSFL is a fat source rich in MCFAs, particularly lauric acid (C12, comprising ∼40 % of total fatty acids) (Suryati et al., 2023).

It was hypothesized that both insect meal and insect oil derived from BSFL could mitigate the negative effects of heat stress on broiler performance due to their antioxidant and immunomodulatory properties. At start of the experiment, no *in vivo* trials have been reported in literature assessing the impact of insect-based ingredients in broiler diets under heat stress conditions.

Therefore, a 2 × 2 × 3 factorial design was used; with and without a heat stress challenge and incorporating insect meal and insect oil at three inclusion levels: 0 %, medium (5 % meal or 2 % oil), and high (10 % meal or 4 % oil). Inclusion levels for insect meal and insect oil align with previous research on insect products under standard conditions (Dabbou et al., 2018; Dörper et al., 2024; Gariglio et al., 2019; Kim et al., 2020).

## Materials and methods

The experiment was conducted from November 16, 2021 until December 21, 2021 in the broiler grower facility of the Cargill Animal Nutrition Innovation Center in Velddriel, The Netherlands. Two different BSFL products (protein and oil) were included at three different levels (zero, medium and high) in both heat-challenged and non-challenged broiler chickens (Table 1). The experimental period was from 0 to 34 d of age. The 12 treatments were randomly distributed per block to 72 pens, resulting in 6 replicates per treatment with 9 broilers each.

**Table 1:** Experimental design.

	Heat stress	BSFL^1^ product	Dietary Inclusion (%)
1	No	Protein	0
2	No	Protein	5
3	No	Protein	10
4	No	Oil	0
5	No	Oil	2
6	No	Oil	4
7	Yes	Protein	0
8	Yes	Protein	5
9	Yes	Protein	10
10	Yes	Oil	0
11	Yes	Oil	2
12	Yes	Oil	4

1 Black soldier fly (*Hermetia illucens*) larvae.

All experimental procedures were approved by the Animal Care and Use Committee of Cargill, Velddriel, The Netherlands (no: AVD2200020198585).

### Diets

Prior to the formulation of diets, batches of corn, wheat, soybean meal, and sunflower seed meal were reserved and chemically analyzed. The dietary formulations were subsequently based on the nutrient composition determined from the analysis of these reserved ingredients. BestMix software was employed to generate the diet formulations, adhering to the guidelines established by the CVB (2018). The control diet was a soybean meal-maize-wheat based diet. Soybean meal was replaced by 10 % inclusion of BSFL protein and soybean oil was partly replaced by 4 % BSFL oil (Table 4). To assure constant nutrient composition between treatments, some ingredients also fluctuated besides soybean meal. By 50/50 mixing of each diet with the basal diet, diets with a 5 % inclusion of BSFL protein and diets with a 2 % inclusion of BSFL oil were produced, respectively. Diets were produced by Research Diet Services (RDS, Wijk bij Duurstede, the Netherlands) and pelleted (starter diets at 2.5 mm, grower diets at 3.0 mm). Diets in each production phase were formulated to be isocaloric and isonitrogenous. A common starter diet (Table 2) was fed to all birds during 0 to 9 d. The chemical composition of BSFL protein and BSFL oil is presented in Table 3 and the experimental grower diets, including the insect products from 9 to 34 d are presented in Table 4. Samples of batches of corn, wheat, soybean meal, and sunflower seed meal prior to production of the diets as well as the experimental diets after pelleting were collected and chemically analyzed for crude protein (Dumas; EC Regulation 152/2009; ISO 16634, 2009), dry matter (EC Regulation 152/2009; ISO 6496, 1999), crude fat (EC Regulation 152/2009; ISO 6492, 1999), ash (EC Regulation 152/2009; ISO 5984, 2002), Ca (ICP MS), and P (EC Regulation 152/2009; ISO 6491:1998). The calculated and analyzed compositions of the experimental diets (including the experimental diets after 50/50 mixing resulting in 5 % inclusion of BSFL protein and 2 % inclusion of BSFL oil) are presented in Table 5. The digestibility coefficients determined by Mwaniki and Kiarie (2018) were used to calculate the standardized ileal digestible (SID) amino acid content for BSFL protein.

**Table 2:** Composition and nutrient levels of starter diet (0 to 9d).

Ingredients (%)	
Soybean meal	36.33
Maize	30.16
Wheat	25.00
Soybean oil	4.60
Limestone	1.74
Calcium carbonate	1.18
Monocalcium phosphate	0.65
Sodium bicarbonate	0.26
Sodium chloride	0.22
Methionine DL	0.29
Lysine HCL	0.18
L-Threonine 98	0.08
Natugrain enzyme	0.010
Natuphos	0.005
Premix^1^	0.500
Nutrients (g/kg)	
Crude ash	58.6
Crude fat	65.7
Crude fibre	28.0
Crude protein	228.7
Calcium	8.8
Chloride	2.0
DEB (meq)	269
Magnesium	1.7
Phosphorus	5.4
Potassium	1.0
Sodium	1.6
Available P	4.0
ME broiler (kcal)	2850
SID Alanine	9.06
SID Arginine	13.74
SID Aspartic acid	19.39
SID Cysteine	2.93
SID Glutamic acid	39.05
SID Glycine	7.97
SID Histidine	5.23
SID Isoleucine	8.49
SID Leucine	15.95
SID Lysine	12.00
SID Meth+Cys	8.80
SID Methionine	5.87
SID Phenylalanine	10.00
SID Proline	12.12
SID Serine	9.75
SID Threonine	7.70
SID Tryptophan	2.42
SID Tyrosine	7.06
SID Valine	9.20

1 Provided per kilogram of diet: 10,000 IU, vitamin A (retinyl acetate); 2,500 IU, vitamin D_3_ (cholecalciferol); 50 mg, vitamin E (dl-a-tocopherylacetate); 2.0 mg, vitamin B_1_ (thiamine); 7.5 mg, vitamin B_2_ (riboflavin); 35.0 mg, vitamin B_3_ (niacin); 3.5 mg, vitamin B_6_ (pyridoxine HCl); 20.0 µg, vitamin B_12_ (cyanocobalamin); 1.5 mg, vitamin K_3_ (bisulfate menadione complex); 12.0 mg, pantothenic acid (d-Ca pantothenate); 1.0 mg, folic acid; 460 mg, choline (choline chloride); 0.2 mg biotin; 0.33 mg, (Na_2_SeO_3_);1.2 mg, (KI); 140 mg, 48 mg, Cu (CuSO_4_.5H_2_O); 265 mg, FeSO_4_.H_2_O); 140 mg, Mn (MnO); 165 mg, (ZnS0_4_.H_2_0); endo-1,4-beta-xylanase EC 3.2.1.8 (1500 EPU); Phytase (600 FTU).

**Table 3:** Chemical composition of BSFL^1^ protein and BSFL^1^ oil.

	BSFL protein	BSFL oil
Nutrients (g/kg DM), Fatty acids (% relative to total fatty acids)		
Crude ash	63.2	-
Crude fat	15.0	990
Crude fibre	90.5	-
Crude protein	560.0	-
SID Alanine	41.3	-
SID Arginine	29.1	-
SID Aspartic acid	50.7	-
SID Cysteine	5.7	-
SID Glutamic acid	60.9	-
SID Glycine	31.6	-
SID Histidine	16.3	-
SID Isoleucine	24.6	-
SID Leucine	40.5	-
SID Lysine	35.5	-
SID Meth+Cys	15.5	-
SID Methionine	9.8	-
SID Phenylalanine	23.3	-
SID Proline	44.2	-
SID Serine	26.0	-
SID Threonine	23.6	-
SID Tryptophan	9.5	-
SID Tyrosine	35.2	-
SID Valine	35.9	-
Capric acid	1.00	0.75
Lauric acid	43.2	36.15
Myristic acid	9.90	8.65
Myristoleic acid	0.20	0.20
Pentadecanoic acid	-	0.05
Palmitic acid	14.7	16.45
Palmitoleic acid	260	2.60
Heptadecanoic acid	0.10	0.20
Cis-10-heptadecenoic acid	-	0.10
Stearic acid	2.40	3.30
Oleic acid	9.00	12.40
Linoleic acid	14.40	15.00
alpha-Linolenic acid	1.20	1.25
Arachidic acid	-	0.40
Arachidonic acid	0.10	0.10
Behenic acid	-	0.15
Trans fatty acid	<0.1	<0.1
Saturated fatty acids	71.5	66.20
Monounsaturated fatty acids	12.6	15.95
Polyunsaturated fatty acids	15.9	16.65
Unsaturated fatty acids	28.5	32.60
Omega-3 fatty acids	1.20	1.25
Omega-6 fatty acids	14.50	15.10
Omega-9 fatty acids	9.10	12.50

1 Black soldier fly (*Hermetia illucens*) larvae.

**Table 4:** Composition and nutrient levels of the basal diet and the two experimental diets with the highest inclusion level of BSFL^1^ protein and BSFL^1^ oil (9 to 34d).

	Basal	10 % BSFL protein	4 % BSFL oil
Ingredients (%)			
Soybean meal	34.22	18.49	34.22
Maize	30.82	33.13	30.82
Wheat	25.00	25.00	25.00
Sunflower meal	0.50	1.76	0.50
Rapeseed meal	0.50	5.00	0.50
BSFL protein	0.00	10.00	0.00
Soybean oil	6.00	3.92	2.00
BSFL oil	0.00	0.00	4.00
Limestone	1.38	1.07	1.38
Monocalcium phosphate	0.26	0.33	0.26
Sodium chloride	0.24	0.24	0.24
Sodium bicarbonate	0.22	0.22	0.22
Methionine DL	0.21	0.19	0.21
Lysine HCL	0.10	0.12	0.10
L-Threonine 98	0.03	0.01	0.03
Natugrain enzyme	0.010	0.010	0.010
Natuphos	0.005	0.005	0.005
Premix^2^	0.500	0.500	0.500
Nutrients (g/kg)			
Dry matter	87.7	88.5	87.7
Crude protein	221.2	221.2	221.2
Crude fat	79.8	73.1	79.8
Crude fibre	29.0	39.9	29.0
Calcium	6.8	6.8	6.8
Phosphorus	4.5	5.2	4.5
Sodium	1.6	1.6	1.6
Potassium	9.7	9.3	9.7
Chloride	2.0	2.0	2.0
DEB (meq)	261	251	261
Available phosphorus	3.1	3.1	3.1
ME broiler (kcal)	2950	2950	2950
SID Alanine	8.84	9.98	8.84
SID Arginine	13.31	11.91	13.31
SID Aspartic acid	18.68	16.86	18.68
SID Cysteine	2.88	2.75	2.88
SID Glutamic acid	38.11	34.55	38.11
SID Glycine	7.77	8.11	7.77
SID Histidine	5.09	4.72	5.09
SID Isoleucine	8.22	8.14	8.22
SID Leucine	15.53	15.27	15.53
SID Lysine	11.00	11.00	11.00
SID Meth+Cys	8.00	8.00	8.00
SID Methionine	5.12	4.95	5.12
SID Phenylalanine	9.70	9.58	9.70
SID Proline	11.87	12.37	11.87
SID Serine	9.45	8.82	9.45
SID Threonine	7.00	7.00	7.00
SID Tryptophan	2.35	1.70	2.35
SID Tyrosine	6.83	9.09	6.83
SID Valine	8.95	9.72	8.95

1 Black soldier fly (*Hermetia illucens*) larvae.

2 Provided per kilogram of diet: 10,000 IU, vitamin A (retinyl acetate); 2,500 IU, vitamin D_3_ (cholecalciferol); 50 mg, vitamin E (dl-a-tocopherylacetate); 2.0 mg, vitamin B_1_ (thiamine); 7.5 mg, vitamin B_2_ (riboflavin); 35.0 mg, vitamin B_3_ (niacin); 3.5 mg, vitamin B_6_ (pyridoxine HCl); 20.0 µg, vitamin B_12_ (cyanocobalamin); 1.5 mg, vitamin K_3_ (bisulfate menadione complex); 12.0 mg, pantothenic acid (d-Ca pantothenate); 1.0 mg, folic acid; 460 mg, choline (choline chloride); 0.2 mg biotin; 0.33 mg, (Na_2_SeO_3_);1.2 mg, (KI); 140 mg, 48 mg, Cu (CuSO_4_.5H_2_O); 265 mg, FeSO_4_.H_2_O); 140 mg, Mn (MnO); 165 mg, (ZnS0_4_.H_2_0); endo-1,4-beta-xylanase EC 3.2.1.8 (1500 EPU); Phytase (600 FTU).

**Table 5:** Calculated and analyzed nutritional composition of the diets (9 to 34d).

Diet phase	Starter diet	Grower diets					
		Basal	5 % BSFL^1^ protein	10 % BSFL^1^ protein	Basal	2 % BSFL^1^ oil	4 % BSFL^1^ oil
Calculated nutrients (g/kg)							
Dry matter	877.0	877.3	881.0	884.7	877.3	877.3	877.3
Crude protein	228.7	221.2	221.2	221.2	221.2	221.2	221.2
Crude fat	65.7	79.8	76.5	73.1	79.8	79.8	79.8
Calcium	8.8	6.8	6.8	6.8	6.8	6.8	6.8
Phosphorus	5.4	4.5	4.9	5.2	4.5	4.5	4.5
Analyzed nutrients (g/kg)							
Dry matter	890.2	885.7	885.8	884.8	883.7	884.1	885.5
Crude protein	228.0	218.0	227.0	221.0	234.0	230.0	226.0
Crude fat	59.0	73.0	74.0	69.0	72.0	73.0	74.0
Calcium	10.7	8.7	7.9	9.3	8.5	9.5	9.6
Phosphorus	6.0	4.8	5.1	5.6	4.8	4.7	5.0
Analyzed vs. Calculated nutrients (%)							
Dry matter	102	101	101	100	101	101	101
Crude protein	98	97	99	99	99	97	98
Crude fat	89	91	97	95	91	92	92
Calcium	121	127	115	137	124	139	141
Phosphorus	111	107	105	107	107	104	111

1 Black soldier fly (*Hermetia illucens*) larvae.

### Animals and housing

The experiment was conducted in an experimental broiler house comprising two rooms, each containing 36 pens. A total of 648 one-day-old male Ross 308 chicks, sourced from 34-week-old broiler breeders, were obtained from a commercial hatchery (Lagerwey, Lunteren, The Netherlands) and randomly assigned to the 72 pens. Each pen housed 9 broilers with an initial average BW of 42.6 g. The pens (90.0 × 112.5 cm) featured a raised floor constructed of stainless steel covered with a 2-cm layer of wood shavings. Each pen was equipped with two sets of two adjustable nipple drinkers and a feeder, which was placed inside the pen for the first 8 days. From d 9 onward, feed was provided via a trough positioned in front of the pen. Both feed and water were provided *ad libitum* throughout the experiment.

Artificial lighting was maintained continuously for 23 hours per day for the first 3 days, then reduced to 20 hours per day from d 4 to 7, and 18 hours per day for the remainder of the experiment. Temperature and ventilation were controlled by a computerized system. In one room, standard temperature program was applied throughout the experiment. Upon arrival, the temperature was set to 34.0°C and was gradually decreased by 0.5°C per day over the first 13 days. From d 14 onwards, the temperature decreased at a rate of approximately 0.4°C per day, reaching a final temperature of 19.6°C by d 34. In contrast, the other room was subjected to heat stress conditions starting on d 9, with the temperature gradually increasing during the day to 32–34°C and at night to 25–28°C. Room temperature was continuously monitored throughout the experiment using a data logger positioned inside the facility.

On d 8, all broilers were spray-vaccinated against Newcastle Disease using the Poulvac NDW vaccine (Zoetis Inc., NJ).

### Growth performance

Group body weights were recorded at the start of the experiment (d 0), while individual body weights were measured on d 9, 21, and 34. Additionally, feed consumption for each pen was recorded on the same days that the broilers were weighed. ADG and ADFI were calculated, and body weight gain : feed intake ratio (G:F) was determined as the ratio of kg of BW gain to kg of feed consumed. The growth performance parameters were corrected for within-period mortality.

### Carcass yield

At 35 d of age, five broilers per pen were randomly selected (excluding obvious outliers). These broilers were then weighed, euthanized using a CO_2_/O_2_ mixture, and bled following an overnight starvation period. The carcass weight was recorded as the total weight of the bird excluding blood, organs, intestines, head, and legs below the hock joint. Carcass yield was calculated as the percentage of the feed-deprived live bird’s weight (measured prior to slaughter). Additionally, the weight of the breast (including the pectoralis major, pectoralis minor, sternum, and clavicle) and the abdominal fat pad was recorded, with their respective yields calculated as a percentage of the carcass weight. For the first two birds, blood (serum), as well as content and tissue samples from the ileum were also collected.

### Blood (serum) collection and analyses

Blood was collected in a single serum tube per bird and allowed to clot at room temperature for 60 minutes. Following clotting, the samples were centrifuged at 1500 g for 10 minutes at room temperature. The resulting serum was carefully pipetted into five 100 µL aliquots and stored at −80°C (transported with dry ice) prior to analysis. C-reactive protein, corticosterone, malondialdehyde, and heat shock protein-70 levels were measured using ELISA kits (manufacturer: MyBioSource, San Diego, California, USA; supplier: Bio-Connect B.V., Huissen, The Netherlands). These biomarkers were selected based on their reported upregulation under heat stress conditions in the literature (Emami et al., 2021; Khondowe et al., 2021; Najafi et al., 2015; Pamok et al., 2009; Tabler et al., 2020; Yin et al., 2021). Additionally, calprotectin levels from ileum samples were also analyzed using ELISA.

### Statistics

Statistical analysis was performed using R version 4.0.2 (R Core Team, 2020). Growth performance parameters were analyzed across the periods: d 0 to 9, d 9 to 21, d 21 to 34, d 9 to 34, and d 0 to 34. Outlier detection for summarized pen data was performed using the standard method, where data points falling outside the range of the quartile ± IQR × multiplier (with the multiplier set to 1.5) were identified as outliers and excluded from the analysis. Mortality was analyzed as a binomial variable. Data are presented as least squares means (LSmeans), and effects were considered significant when *P* ≤ 0.05.

The statistical model used in this experiment was:

Yijk=μ+αi+βj+γk+αβij+αγik+βγjk+αβγijk+Cl+εijk

Where:

Y_ijk_ = *A* specific trait per experimental unit

μ = Overall mean

α_i_ = Heat stress effect (*i* = 1-2)

β_j_ = Insect product effect (*j* = 1-2)

δ_k_ = Inclusion level effect (*k* = 1-3)

αβ_ij_ = Interaction effect between heat stress and insect product

αδ_ik_ = Interaction effect between heat stress and inclusion level

βδ_jk_ = Interaction effect between insect product and inclusion level

αβδ_ijk_ = Interaction effect between heat stress, insect product and inclusion level

Cl = Block effect (*l* = 1 – 6)

ε_ijk_ = Error term

## Results

The nutritional composition of the diets was generally in accordance with the expected values (Table 5), with the exception of calcium levels. The deviation in calcium concentrations observed in the ICP results has been noted in previous trials and is likely attributable to the methodology used. Given that the calcium levels were consistently higher than expected across all treatments, this discrepancy is unlikely to have influenced the trial outcomes. The health status of the birds was monitored throughout the experiment, and no significant issues were observed. Mortality from 0 to 34 d of age was 0.6 %. When including culling, the total mortality reached 2.0 %, which is considered low compared to general farm practices, where typical mortality rates range from 3 to 4 %.

**Fig. 1:**
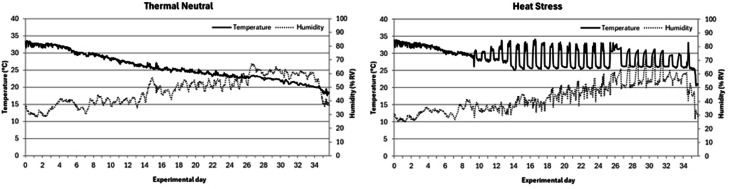
Realized room temperature and relative humidity in standard temperature conditions (left) and heat stress conditions (right) during the experimental period.

The realized temperature and relative humidity in the experimental rooms (0 to 34 d of age) is presented in Fig. 1. It is shown that after 9 d of age in the heat stress room cyclic heat stress was induced by a rise in temperature during the day to 32–34°C and at night to 25–28°C until d 34 while in the control room the temperature decreased gradually, reaching a final temperature of 19.6°C by d 34.

### Effect of heat stress on growth performance

The experimental period commenced on d 9, with the introduction of the experimental diets and the onset of cyclic heat stress. At the start of the experiment, the broilers had an initial BW of 42.5 g, and at 9 d of age, the BW was 255 g. Over the period from 0 to 9 d of age, ADFI was 226 g, the G:F ratio was 0.949, and the mortality including culling rate was 0.9 %. Growth performance and carcass data throughout the experimental period and P-values for the main effects and interaction effects are summarized in Table 6 and Table 7, respectively.

**Table 6:** Performance and carcass characteristics of Ross 308 broilers (0 to 34 d) by heat stress challenge, BSFL^1^ product, and inclusion level groups.

Heat stress	No		No		No		No		No		No		Yes		Yes		Yes		Yes		Yes		Yes	
BSFL product	Protein		Protein		Protein		Oil		Oil		Oil		Protein		Protein		Protein		Oil		Oil		Oil	
Inclusion (%)	0		5		10		0		2		4		0		5		10		0		2		4	
ADFI (g/d)																								
d 9-21	73.0		77.9		71.3		73.6		72.7		74.1		73.1		70.8		73.1		71.8		72.3		72.8	
d 21-34	155.3	^abc^	157.8	^a^	147.2	^abc^	156.4	^ab^	145.2	^abc^	154.1	^abc^	146.7	^abc^	142.6	^c^	142.5	^c^	143.1	^bc^	145.9	^abc^	145.0	^abc^
d 9-34	115.8	^bc^	119.5	^b^	110.8	^bc^	116.6	^bc^	110.2	^bc^	115.6	^bc^	111.4	^bc^	108.1	^c^	108.1	^c^	108.8	^c^	110.6	^bc^	110.3	^bc^
d 0-34	91.6	^bc^	94.4	^b^	87.9	^bc^	91.8	^bc^	87.4	^bc^	91.6	^bc^	88.6	^bc^	85.6	^c^	85.8	^c^	86.2	^c^	88.1	^bc^	87.6	^bc^
ADG (g/d)																								
d 9-21	57.4	^ab^	61.1	^a^	54.5	^b^	59.2	^ab^	56.0	^ab^	59.3	^ab^	57.8	^ab^	56.5	^ab^	55.8	^ab^	56.9	^ab^	58.0	^ab^	57.4	^ab^
d 21-34	110.1		111.2		104.9		111.5		106.9		108.5		105.8		102.5		101.2		102.6		106.9		106.0	
d 9-34	84.8	^ab^	87.1	^a^	80.7	^ab^	86.3	^ab^	83.2	^ab^	84.8	^ab^	82.7	^ab^	80.4	^ab^	79.2	^b^	80.6	^ab^	83.4	^ab^	82.7	^ab^
d 0-34	68.6		70.4		65.5		69.2		67.1		68.5		67.2		65.0		64.3		65.3		67.7		67.0	
G:F (g/g)																								
d 9-21	0.785		0.784		0.765		0.803		0.796		0.793		0.790		0.799		0.782		0.792		0.803		0.789	
d 21-34	0.709		0.705		0.713		0.713		0.722		0.704		0.721		0.718		0.711		0.717		0.732		0.731	
d 9-34	0.732		0.729		0.729		0.740		0.745		0.734		0.743		0.744		0.734		0.741		0.755		0.749	
d 0-34	0.749		0.745		0.746		0.754		0.760		0.748		0.759		0.759		0.750		0.757		0.769		0.764	
Mortality																								
d 0-34	0.000		0.019		0.000		0.019		0.000		0.000		0.000		0.037		0.019		0.000		0.000		0.019	
BW (g)																								
d 9	254.8		257.2		253.4		257.4		252.0		252.4		259.5		254.6		252.3		244.9		258.7		259.9	
d 21	943.3		995.5		907.9		963.1		945.8		963.9		953.0		933.2		921.9		934.6		955.3		948.9	
d 34	2374.2	^ab^	2441.5	^a^	2271.8	^ab^	2412.0	^ab^	2335.1	^ab^	2385.7	^ab^	2348.0	^ab^	2265.4	^ab^	2239.0	^b^	2282.7	^ab^	2345.0	^ab^	2326.4	^ab^
Carcass yields																								
BW (kg)	2.51		2.62		2.49		2.53		2.49		2.56		2.48		2.43		2.40		2.47		2.53		2.50	
Carcass (%)	76.19		76.36		76.43		76.19		76.21		76.38		78.24		77.36		77.21		77.34		78.41		77.59	
Breast (%)	33.48		33.04		32.90		33.81		32.95		33.41		31.78		32.12		31.84		31.96		32.26		32.31	
Fat pad (%)	0.89		0.99		1.08		0.83		0.96		0.99		1.22		1.29		1.29		1.17		1.36		1.21	

ADFI, ADG, and G:F were corrected for within-period mortality.

Carcass yields presented as percentage of live body weight and breast and fat pad presented as percentage of carcass weight.

1 Black soldier fly (*Hermetia illucens*) larvae.

**Table 7:** P-values for growth performance and carcass data for treatments Heat stress challenge, BSFL^1^ product, and Inclusion level.

Experimental treatment	Heat stress	BSFL product	Inclusion	Heat stress * BSFL product	Heat stress * Inclusion	BSFL product * Inclusion	Heat stress * BSFL product * Inclusion
ADFI (g/d)							
d 9-21	0.234	0.716	0.858	0.759	0.906	0.405	0.068
d 21-34	**0.011**	0.790	0.212	0.453	0.491	**0.050**	**0.011**
d 9-34	**0.015**	0.840	0.409	0.421	0.756	0.064	**0.020**
d 0-34	**0.022**	0.828	0.520	0.386	0.858	0.080	**0.020**
ADG (g/d)							
d 9-21	0.259	0.403	0.361	0.862	0.871	**0.025**	**0.012**
d 21-34	0.055	0.281	0.160	0.375	0.389	0.109	**0.036**
d 9-34	0.063	0.229	0.158	0.598	0.598	0.074	**0.035**
d 0-34	0.081	0.364	0.248	0.468	0.739	0.086	**0.041**
G:F (g/g)							
d 9-21	0.083	**<0.0001**	**0.001**	**0.007**	0.096	0.316	0.545
d 21-34	**0.047**	**0.033**	0.427	0.409	0.863	0.158	0.054
d 9-34	**0.027**	**0.002**	0.129	0.759	0.639	0.288	0.259
d 0-34	**0.000**	**0.004**	0.123	0.991	0.638	0.242	0.209
Mortality							
d 0-34	1.000	1.000	1.000	1.000	1.000	1.000	1.000
BW (g)							
d 9	0.857	0.673	0.880	0.962	0.469	0.316	0.056
d 21	0.296	0.413	0.305	0.945	0.648	0.134	0.103
d 34	**0.050**	0.289	0.186	0.683	0.775	0.071	**0.035**
Carcass yields							
BW (kg)	0.121	0.330	0.646	0.166	0.915	0.324	0.153
Carcass (%)	**0.009**	0.822	0.839	0.626	0.528	0.338	0.237
Breast (%)	**0.006**	0.162	0.729	0.967	0.091	0.570	0.905
Fat pad (%)	**<0.0001**	0.140	**0.000**	0.526	0.078	0.239	0.771

ADFI, ADG, and G:F were corrected for within-period mortality.

1 Black soldier fly (*Hermetia illucens*) larvae.

Cyclic heat stress was applied from 9 d of age. Although there was a slight increase in the number of birds culled under heat stress conditions, no significant differences were found in mortality rates (Table 7). Additionally, the birds exhibited visible signs of heat stress, such as panting behavior. From d 9 to 21, no significant differences in ADFI were observed. Between d 21 and 34, ADFI in the heat stress room was significantly lower (*P* ≤ 0.011), while ADG remained comparable between the heat stress and control rooms. As a result, the birds in the heat stress room demonstrated a significantly improved G:F ratio (*P* = 0.047) between d 21 and 34. Throughout the experimental period, exposure to heat stress resulted in a significant reduction in ADFI with a lower body weight by 2.9 % on 34 d of age (*P* = 0.050). Heat stress resulted in a significant reduction in relative breast yield, alongside an increase in relative fat pad (Table 6). These carcass effects were consistent across the heat-stressed group.

### Effect of heat stress on stress biomarkers

Heat stress had measurable effects on stress biomarkers at d 35, as presented in Table 8. Ileum calprotectin levels were significantly elevated in broilers exposed to heat stress compared to those in neutral climate conditions (*P* = 0.002). Additionally, serum corticosterone levels showed a tendency to decrease in broilers under heat stress conditions (*P* = 0.085). The other stress biomarkers were not affected by heat stress and no interaction between heat stress and insect products has been observed for stress biomarkers.

**Table 8:** Effects of heat stress challenge on biomarkers in Ross 308 broilers at 35 d of age.

Heat stress	No		Yes		SEM	P-value
Heat shock protein-70 (ng/mL)	0.92		0.96		0.31	0.934
Corticosterone (ng/mL)	1.70		0.69		0.31	0.085
C-reactive protein (ng/mL)	1067.1		865.7		236.7	0.580
Malondialdehyde (µM)	283.1		321.8		43.3	0.562
Calprotectin (ng/mL)	81.9	^b^	124.0	^a^	9.3	**0.002**

### Effect of insect products on growth performance

In general, no clear effects of insect products on performance were observed. However, several interaction effects between insect products, inclusion level, and heat stress were identified (Table 7). Significant three-way interaction effects were detected between heat stress, insect product, and inclusion level after 21 d of age for ADFI, ADG, and BW d 34. Under thermoneutral conditions, ADFI and ADG decreased numerically at 2 % BSFL oil inclusion and increased numerically at 4 % BSFL oil inclusion relative to the 2 % inclusion level. In contrast also under thermoneutral conditions, 5 % BSFL protein inclusion led to a numeric increase in ADFI and ADG and led a numerically reduction in ADFI and ADG at 10 % BSFL protein inclusion. The effects on ADFI also influenced BW on d 34 in a similar manner. A significant three-way interaction effect on BW d 34 was observed (*P* = 0.035). Under heat stress conditions, BSFL oil inclusion resulted in a numerically increase in BW, whereas increasing levels of BSFL protein led to a numerically decrease in BW. In thermoneutral conditions, BW increased with 5 % BSFL protein inclusion, but decreased numerically when the inclusion level was raised to 10 %. For BSFL oil, BW decreased numerically with 2 % inclusion, followed by a numerically increase with the 4 % inclusion level relative to the 2 % inclusion level.

## Discussion

### Effect of heat stress on growth performance

The improvement in G:F ratio is contradictory to findings of Teyssier et al. (2022) who showed a 20 % reduction in BW gain and 10 % lower G:F ratio at cyclic heat stress (35°C for 12 h daily and reduced to 24°C each night) from 20 d of age. This suggests that starting cyclic heat stress at an older age may have impacted growth performance results more than in our experiment, in which cyclic heat stress started at 9 d of age. The observed reduction in final BW of 2.9 % was substantially smaller than the reduction reported by Teyssier et al. (2022). Nutrient density reported by Teyssier et al. (2022) was higher than in our experiment which may have increased the metabolic pressure of the broilers subjected to heat stress. Together, these findings highlight how both age at onset and diet composition modulate the growth response to heat stress. The reduction in relative breast yield and increase in fat pad are commonly observed in heat-stressed broilers. Reduced protein synthesis and adaptive energy storage as fat may explain these effects (Maharjan et al., 2020) (El-Tarabany et al., 2021). This aligns with the broader literature showing that carcass composition is a sensitive indicator of metabolic adjustments under heat stress.

### Effect of heat stress on stress biomarkers

the elevation in ileum calprotectin under heat stress is consistent with dal pont, et al. (2021), who reported an increase in fecal calprotectin in broilers subjected to chronic stress, indicating chronic gut inflammation. The tendency for corticosterone to decrease under heat stress contrasts with Khondowe, et al. (2021), who observed increased corticosterone levels. The reason for the contrasting results remain unclear, but may relate to differences in heat stress models or bird genotypes. These results suggest that not all biomarkers respond consistently across experimental conditions.

### Effect of insect products on growth performance

The reduction in BW associated with BSFL protein was observed in both neutral and heat stress conditions. Several hypotheses are possible as reason for this effect. The amino acid content of the diets was analyzed to ensure they were isonitrogenous, and no deviations were found. Another hypothesis suggested that the digestibility value of BSFL protein may have been overestimated. There is no clear consensus in the literature regarding the maximum inclusion rate of BSFL protein and its potential limitations. In a study by Heuel, et al. (2022), BSFL protein was included at up to 20 %, demonstrating superior performance compared to soybean protein. Conversely, Kim, et al. (2022) concluded that BSFL protein could be included at 7.5 %, but inclusion at 15 % resulted in detrimental effects on growth performance and health in Ross 308 male broilers. It is hypothesized that chitin, a component of insect exoskeletons, may play a role in these outcomes, though limited literature is available on its nutritional and antinutritional properties. Chitin and chitosan, polysaccharides found in high concentrations in insects, are known to be indigestible by most animals due to the lack of chitinase in the gastrointestinal tract. Chitin and its derivatives have been reported to positively influence animal health, with chitosan exhibiting antibacterial properties against bacteria, yeasts, and fungi (Dörper et al., 2021). Additionally, the diet containing 10 % BSFL protein included 5 % rapeseed meal to meet nutritional requirements. Since rapeseed meal is commonly used in broiler diets at higher inclusion levels, it is not expected to have contributed to the observed reductions in ADFI and BW.

This trial demonstrates that replacing traditional ingredients with BSFL-based products is feasible under both neutral and heat stress climate conditions. Under mild cyclic heat stress, BSFL protein (5-10 %) and oil (2-4 %) did not statistically improve growth performance, but numerical benefits of BSFL oil were observed which warrant further validation under higher stress intensity in Ross 308 broilers. No interaction between heat stress and insect products has been observed for stress biomarkers. The mild heat stress model may mask the effects of BSFL products, and it is recommended to verify under moderate to severe heat stress conditions in the future.

## CRediT authorship contribution statement

**T. Veldkamp:** Writing – original draft, Supervision, Project administration, Investigation, Funding acquisition, Conceptualization. **A. Rezaei Far:** Writing – original draft, Methodology, Investigation, Formal analysis, Data curation, Conceptualization. **J.J. Mes:** Writing – review & editing, Resources, Methodology, Investigation, Data curation, Conceptualization. **S. Naser El Deen:** Writing – review & editing, Methodology, Investigation, Data curation. **P.G. van Wikselaar:** Writing – review & editing, Methodology, Investigation, Formal analysis, Data curation. **I. Fodor:** Writing – review & editing, Software, Methodology, Investigation, Formal analysis, Data curation, Conceptualization. **H. Chen:** Writing – review & editing, Resources, Methodology, Investigation, Formal analysis, Data curation, Conceptualization.

## Disclosures

The authors declare that they have no known competing financial interests or personal relationships that could have appeared to influence the work reported in this paper.
